# The COVID-19 infection in children and its association with the immune system, prenatal stress, and neurological complications

**DOI:** 10.7150/ijbs.66906

**Published:** 2022-01-01

**Authors:** Suliman Khan, Rabeea Siddique, Xiao Hao, Yueting Lin, Yuxin Liu, Xiaoyan Wang, Linlin Hua, Ghulam Nabi

**Affiliations:** 1 Department of Cerebrovascular Diseases, The Second Affiliated Hospital of Zhengzhou University, Zhengzhou, China; 2 Department of Medical Lab Technology, The University of Haripur, Pakistan; 3 Advanced Medical Center, The Second Affiliated Hospital of Zhengzhou University, Zhengzhou, China; 4 The Second Affiliated Hospital of Zhengzhou University, Zhengzhou, China; 5 Department of Child Healthcare, Hubei Maternal and Children's Hospital, Wuhan, 430070, China; 6 Ministry of Education Key Laboratory of Molecular and Cellular Biology, Key Laboratory of Animal Physiology, Biochemistry and Molecular Biology of Hebei Province, College of Life Sciences, Hebei Normal University, Shijiazhuang, 050024, China

## Abstract

The Coronavirus disease 2019 (COVID-19)” caused by the “severe acute respiratory syndrome corona virus 2 (SARS-CoV-2)” has caused huge losses to the world due to the unavailability of effective treatment options. It is now a serious threat to humans as it causes severe respiratory disease, neurological complications, and other associated problems. Although COVID-19 generally causes mild and recoverable symptoms in children, it can cause serious severe symptoms and death causing complications. Most importantly, SARS-CoV-2 can cause neurological complications in children, such as shortness of breath, myalgia, stroke, and encephalopathy. These problems are highly linked with cytokine storm and proinflammatory responses, which can alter the physiology of the blood-brain barrier and allow the virus to enter the brain. Despite the direct infection caused by the virus entry into the brain, these neurological complications can result from indirect means such as severe immune responses. This review discusses viral transmission, transport to the brain, the associated prenatal stress, and neurological and/or immunological complications in children.

## Introduction

SARS-CoV-2 causes COVID-19, which has killed a large number of people across the globe. It is an enveloped, positive-sense, single-stranded RNA virus having a size of approximately 30 kb. Although COVID-19 has been reported to harm adults more than children, its severity has also been widely reported in children 1. Interestingly, the development of COVID-19 is the same in all individuals regardless of the severity of disease, and age of the infected individual. The disease starts with the entrance of the virus into host cells, which then causes symptoms. The primary symptoms (pneumonia, cough, fatigue) of COVID-19 are associated with the pulmonary system, it is now considered to affect multiple organ systems, especially, the brain, by causing neurological complications in approximately, 36% of the total COVID-19 patients. Earlier studies have reported that neurological consequences were also caused by Severe Acute Respiratory Syndrome (SARS) and Middle East Respiratory Syndrome (MERS), indicating that all human coronaviruses induce neurologic complications. However, neurological complications caused by COVID-19 are at a higher extent than that of SARS and MERS. It is unclear how COVID-19 induces these neurological complications. One possible reason could be the direct infection of the brain by the virus as SARS-CoV-2 has been reported to invade the brain and might infect the peripheral and central nervous systems 2.

Growing evidence indicates that COVID-19 impacts both the central nervous system (CNS) and peripheral nervous system (PNS) to cause respective complications due to either direct infectiousness or immune-mediated disease in response to COVID-19 infection. In addition, histopathological changes such as CNS infarction due to cerebral thromboembolism and viral RNA in CNS further indicate the impact of SARS-COV-2 on CNS alterations 3. Reportedly, patients with COVID-19 are diagnosed with taste and smell dysfunction, nausea, and headache and are prone to develop severe neurological complications including encephalopathies, Myelitis, Rhabdomyolysis, Guillain-Barré syndrome, cognitive syndrome, affective disorder, and cerebrovascular complications such as strokes, intracerebral haemorrhages, and CNS vasculitis) 4. However, these neurological complications are presented differently based on underlying comorbidities and age 1.

The entry of SARS-CoV-2 to human host cells requires the cellular receptor angiotensin‐converting enzyme 2 (ACE2) and serine protease TMPRSS2 for spike (S) protein priming 5. After entry, the virus can multiply and disseminate into the airway after shutting down IFN type 1 antiviral pathway. The virus generally spreads from the lung and can also disseminate to other ACE2 expressing tissues 6. It is widely known that ACE2 is highly abundant on alveolar pneumocytes, enabling SARS-CoV-2 to enter and infect these cells. Similarly, evidence indicates that ACE2 is also expressed on neuronal and glial cells in the human CNS 7, suggesting that SARS-CoV-2 can enter these cells. Although, the presence of SARS-CoV-2 RNA in CNS regions has been confirmed 3, the route of viral transport to these regions and the underlying mechanisms are yet to be investigated. Preliminary evidence indicates that neuro-invasion of SARS-CoV-2 can occur via regional nervous structures at the neural-mucosal interface 8, suggesting that the possible transport of the virus along the olfactory tract of the CNS may be the reason for neurological complications. However, Meinhardt et al. 3 reported that there is no direct connection between viral RNA in CNS and the olfactory mucosa, indicating that the virus entry to CNS may involve other routes/mechanisms such as CNS endothelia, axonal transport, and leukocytes 8.

In this paper, we discuss the infection of COVID-19, its transmission, and associated neurological consequences in children. We also highlight the possible mechanisms associated with viral transport to CNS and increased neurological disorders risk. This review will help readers and researchers to understand the nature of COVID-19 in children and how the associated complications can be managed or prevented.

## COVID-19 and the associated risk of respiratory and neurological complications in children

The currently ongoing pandemic of COVID-19 has a wide-range impact on all age groups, including children. It is now evident that COVID-19's common clinical manifestations in children include fever and cough. These symptoms generally develop atypically relative to adults. In children, the disease can be from asymptomatic to severe illness and life-risking; however, severe illness is not common because children are less likely to have underlying diseases including hypertension, diabetes, cardiovascular problems. Moreover, conditions such as differential expression of ACE2, co-morbidities, and predisposition to pro-inflammatory states can impact viral entry, replication, inflammation, hypoxia, and tissue injury. It is further aided by the higher efficiency of the innate immune response, which typically declines with age. 9.

The COVID-19 can pose a life-threatening risk in infants; therefore, serious attention is needed. Studies have shown that fever is the most common feature of COVID-19, while rhinorrhea, cough, gastrointestinal symptoms, headache, encephalopathy, mild shortness of breath, and myalgia have also been reported in children. Interestingly, in some asymptomatic cases, ground-glass opacities and mild bronchial thickening can be evident. However, some reports have indicated that children infected with SARS-CoV-2 may not present clinical or radiological features 9. A recent report has detailed the clinical features such that children with COVID-19 were presented with fever (64%), cough (35%), and rhinorrhea (16%), while 15% of them were asymptomatic. Overall, 54% of the cases presented ground-like opacities in radiological investigations, while the common findings from laboratory tests included elevated D-dimer (52%), C-reactive protein (40%), and lymphopenia (33%). Overall, 15% of these patients, where the majority required intensive care developed multisystem inflammatory syndrome manifesting with marked elevated inflammatory biomarkers, gastrointestinal symptoms, shock, and left ventricular systolic dysfunction. Nevertheless, children may develop severe symptoms. Therefore, early detection of COVID-19 and timely diagnosis of the multisystem inflammatory syndrome are necessary for proper management and prevention of transmission 10.

Despite multisystem inflammatory syndrome and respiratory symptoms, neurological complications have also been associated with COVID-19 in children, such as headaches, encephalopathy, and altered mental status. Moreover, children diagnosed with multisystem inflammatory syndrome suffered from severe neurological aberrations, including encephalitis, seizure, coma, demyelinating disorders, dysgeusia or ageusia, aseptic meningitis, stroke dysarthria, dysphagia, cerebellar ataxia, axial hypotonia, drowsiness, or moaning, and peripheral neuropathy. In COVID-19 infected children presented with fever, shock, and rash, MRI or CT changes involved splenium of the corpus callosum, which may increase the risk of Kawasaki or alike disease, as well as inflammatory encephalopathies 1. However, further studies are required to verify the actual neurological conditions developed by the aforementioned conditions.

## The cellular invasion and transmission processes of the virus in Children

The source of emergence and zoonotic transmission is still debatable; however, human-to-human transmission has been confirmed widely. It is now evident that children can easily acquire SARS-CoV-2 from an adult through direct contact. However, the transmission rate from children is comparatively low. Although SARS-CoV-2 RNA has been detected in the stool samples of pediatric patients, there is no convincing evidence indicating fecal-oral transmission of the virus. Among the various transmission media, droplets are considered the most important medium of transmission in children 9. The entry process of SARS-CoV-2 into the cell requires spike (S) glycoprotein, which facilitates receptor (ACE2) binding. This S protein is displayed in the form of trimers that give rise to the coronated appearance of the virus. The S protein is comprised of S1 and S2 subunits, where S1 is responsible for ACE-2 binding, while the latter subunit is required for cellular fusion 11-13. The entry process starts with the interaction between ACE2 and receptor-binding domain (RBD) within S1, followed by the cleavage process mediated by proteases. Previous studies have reported that S protein is a key player in the process of cellular entry, and thus can influence the transmission process. The entry process is highly affected by a number of mutations in S protein, such as the substitution of aspartic acid to glycine at amino acid position 614 (D614G). Earlier reports have demonstrated phenotypic advantage conferred by D614G 14, however, the mechanism underlying this enhancement is yet to be investigated. Interestingly, this crucial role of S protein can be utilized to develop a vaccine, such that recombinant S protein can trigger immune responses, which may help in treating and preventing COVID-19 15. Patra et al 16 reported that S protein facilitates angiotensin II-mediated signaling cascade influences transcriptional regulatory molecules via MAPK activation, and increases the release of IL-6. Moreover, STAT3 phosphorylation at the Tyr705 residue was found to play a crucial role as a transcriptional inducer for the expression of MCP-1 and SOCS3. Overall, the presence of S protein promotes IL-6 trans-signaling by AT1 axis activation to initiate hyper-inflammatory responses. Considering the infection in neonates, several reports have outlined the adverse outcomes of COVID-19 during pregnancy, however, none of the reports has confirmed vertical transmission. We have previously reported that SARS-CoV-2 infection can increase the risk of neonatal pneumonia and preterm delivery. We noticed that all neonates except two suspected cases delivered by women infected with COVID-19 were healthy. These results suggest that the intrauterine vertical transmission may not occur, however, SARS-CoV-2 can lead to adverse pregnancy outcomes 17. In the case of natural birth, we found similar results to those of a Cesarean section. Overall, these reports did not find convincing evidence regarding vertical transmission or maternal-to-neonatal intrapartum transmission of COVID-19 18. In contrast, Peng et al. reported the possibility of vertical transmission into pre-term twin neonates from an infected mother. In this report, twin neonates were tested positive for COVID-19 infection soon after their delivery, and this infection was suggested to be caused by intrauterine vertical transmission. However, this study had some crucial limitations such as amniotic fluid and cord blood were not tested, the sample size was small, and detailed laboratory testing was not available 19. As per the literature search, none of the studies reported convincing evidence 20. Therefore, we conclude that there is minimal possibility of vertical transmission of SARS-CoV-2 from infected mothers into infants. Based on these indications, it is recommended that mothers continue breastfeeding their infants with proper care and following the hygiene measures 9.

### COVID-19 during pregnancy and the possible mental complications in neonates

The COVID-19 is profoundly affecting mental health in infected individuals and increasing the risk of psychological problems in non-infected individuals, especially in pregnant women and children. In response to the global pandemic, individuals develop anxiety, anger, fear, and disturbed wake and sleep routines that may alter the hormonal balance by affecting the hypothalamic-pituitary-gonadal axis. These problems may cause adverse pregnancy and neonatal outcomes, such as the increased risk of schizophrenia in neonates. Maternal psychosocial stress can adversely affect the development and maturation of the fetal brain in response to elevated maternal cortisol and cytokines levels and disrupted serotonin homeostasis. This can also affect the vaginal ecosystem and microbiome, which induces disruption of the fetal gut-brain axis, thereby developing neurodevelopmental disorders in growing children. Maternal psychosocial stress can also increase susceptibility to several medical conditions such as smaller head circumference, low birth weight, preterm birth, morphological, and physiological alteration in the fetal brain, and neurobehavioral disorders (schizophrenia, autism, learning disorders, and mood disorders) 21.

We know that normal daily routines and mental health are inevitable to maintain proper circadian rhythms. However, in the current scenario of the COVID-19 pandemic, individuals are restricted from outdoor activities, which affect their daily schedules, such as sleep-wake timing and eating routines. Thus, the chances of rhythm disruption are increased. Exposure to light at night and disturbed sleep patterns in children induces perturbation of the circadian rhythm, leading to mood disorders, anxiety, and sleep disorders. Moreover, limited exposure to the outdoor environment and higher access to electronic devices may increase children's risk of severe mental illnesses 22. In addition, the current situation of the COVID-19 pandemic may be challenging for children with disabilities, existing mental health issues, and trauma experiences. Therefore, treatment is necessary for mitigating long-term consequences 23. Based on these indications, ignoring psychological outcomes in pregnant women during the current pandemic, ensued by troubled sleeping and biological functioning, may lead to serious medical complications. Moreover, researchers and practitioners must perform proper investigations to detect specific psychological problems in children to provide medical assistance. Nevertheless, timely psychological interventions are needed to mitigate the risk of maternal and neonatal mental health problems in response to the current situation of the COVID-19 pandemic.

## COVID-19 mediated prenatal maternal stress and childhood outcome

COVID-19 is a significant traumatic experience, particularly during sensitive time windows such as pregnancy and neonatal life, altering the mother's psychosocial well-being and therefore, altering the fetal neural programming 21. In humans and animal models, the relationship between prenatal maternal stress and childhood outcomes is sex-dependent 24. For example, in rodents, relative to females, males have stronger associations with postnatal outcomes, including altered brain development and behavior 25. Similarly, in humans, the prevalence of certain prenatal stress-linked disorders, including attention deficit hyperactivity disorders, schizophrenia, and autism spectrum disorders, has a notable sex difference 26-28. Some prenatal stress-linked sex differences in offspring outcomes persist into later childhood. Prenatally stress-exposed female offspring have a poorer motor function at age 5 ^½^ years, unlike males 29. Similarly, a positive correlation was reported between prenatal stress, and greater right amygdala volume, and altered neural activity and connectivity in girls but not in boys in childhood 30,31. Prenatal stress is also associated with an adverse effect on a child's cognitive development in a sex-dependent manner, affecting girls more than boys 32. Unlike girls, the emotional-behavioral problems among boys are linked with prenatal stress 33. Sutherland and Brunwasser 34 concluded that girls exposed to prenatal stress are at high risk of developing anxiety and affective disorders. In our view, these findings indicate that biological sex should also be considered when designing and evaluating prenatal maternal stress studies. These few pieces of evidence indicate that different sexes may respond differently to prenatal stress and that the effects may also differ at different stages of development 24. Possibly, the stress could affect the prenatal hormonal milieu and sex-dependent development. However, the underlying mechanism is not explored yet. Furthermore, studies are needed to understand a possible link between prenatal stress and multi-transgenerational risks through epigenetic programming.

## COVID-19 mediated neurological complications in children

Recent reports have indicated several neurologic complications in individuals infected with COVID-19. They commonly develop sensory deficits in taste and smell, peripheral nervous system disorders, strokes, delirium, headaches, and encephalopathy. Although, COVID-19-associated neurological manifestations among children have not been reported widely, however, reports of neurologic dysfunction in neonates and children are increasing with time. Despite affecting the pulmonary system, gastrointestinal tract, kidneys, liver, and heart, COVID-19 has been recognized to affect the peripheral and central nervous systems.

**Cerebrovascular Disease:** COVID-19 mediated inflammation of the vascular epithelium can increase the risk of ischemic strokes, affecting individuals with large vessel occlusions (ischemic stroke). In children with COVID-19, stroke has not been reported; however, with the growing variants of SARS-CoV-2 such as delta virus, there is a risk of serious consequences.

**Encephalitis:** Although some of the COVID-19 patients have been diagnosed with encephalitis, its occurrence remains controversial. In recent investigations, research failed to detect the virus in cerebrospinal fluid. In addition, based on pediatric autopsy cases, no neuropathological conditions have been reported yet. However, a wide range of studies is needed to verify the association of COVID-19 with encephalitis in pediatric patients.

**Seizures:** Seizures have been reported in COVID-19 patients. The occurrence rate is very low; therefore, we cannot conclude if the infected patients are at higher risk. However, it cannot be ignored as the newly emerging variants of SARS-CoV-2 are more aggressive. Therefore, seizures and the associated neurological symptoms may occur at a higher rate in the near future. However, we are not sure if the lower occurrence of seizures has been accurately reported, as encephalopathy is a frequent complication of COVID-19. Thus, further studies should carefully consider the seizures while investigating COVID-19 patients.

**Hyposmia:** Hyposmia is the most reported symptom associated with the peripheral nervous system. It is thought that neural mechanism is involved in COVID-19 hyposmia, as decreased smell occurs even in the absence of significant mucosal congestion or local inflammation. Loss of smell and taste are not common in children, but we cannot rule out the possibility that these do not occur in children.

**Demyelinating Disorders:** COVID-19 increases the risk of Guillain-Barré syndrome in children, indicating the occurrence of post-infectious autoimmune responses against the peripheral nervous system. According to the previous reports, it is possible that the virus does not cause neurological symptoms directly but indirectly through cytokines involvement, hypoxia, and additional relevant phenomena.

## Mechanism of neurological involvement

In CNS, ACE2 receptors present on vascular endothelial cells determine the mechanism of action for SARS-CoV-2 infection. The binding of the virus with ACE2 is thought to trigger pro-coagulable and pro-inflammatory reactions by disrupting vascular integrity and clotting cascade activation. Moreover, this interaction between virus and ACE2 may disrupt the blood pressure autoregulation. These events ultimately lead to the occurrence of neurological complications, including ischemic stroke 1. Although the bases of neurovirulence at the molecular level have not been manifested yet, the potential mechanisms underlying this phenomenon may be either the direct neurotropic effect of the virus, SARS-CoV-2 mediated inflammatory responses, prothrombotic effect on the CNS vasculature, and autoimmune effect mediated by the immune system. Nevertheless, it is important to unveil the underlying mechanisms in order to develop promising therapeutic options. Among the proposed routes of entry, olfactory sensory neurons are considered crucial, which cross the cribriform plate into the olfactory bulb, and then travel to the brain stem, thalamus, basal ganglia through cranial nerve I (olfactory nerve). However, this possibility still needs to be proven as data are scarce to prove SARS-CoV-2 infects olfactory sensory neurons. Despite the lack of convincing evidence, hyposmia can be considered evidence to justify that the olfactory epithelium is a potential site of viral entry into the brain. Interestingly, considering the taste loss, it is possible that the virus enters the brain via the tongue's sensory system through cranial nerves. Then, it could reach CNS via cranial nerve V from corneal epithelium or buccal epithelium, which might be the reason for hypogeusia and altered vision.

Researchers have suggested that the bloodstream or disruption of the BBB might be another potential route for viral transport to the brain. It is thought the virus may travel through the systematic and cerebral circulation to damage the capillary epithelium. However, no convincing evidence is available to confirm that the virus produces sustained or significant viremia. We know that for molecular transport into the brain, BBB is required. Hence attachment of the virus to ACE-2 on BBB facilitates its transport into the brain or CNS, leading to edema and endothelial damage. In response to COVID-19 mediated inflammatory response BBB may get disrupted and become dysfunctional, enhancing viral transmission or activating immune cells to the CNS. Inflammation is exacerbated by the inflammatory cytokines produced by neural mast cells and activated glia cells, which induce the viral flow through lymphatic channels that may breach the barrier between blood and CSF, and as a result, SARS-CoV-2 enters the brain. However, there is no convincing evidence regarding the presence of the virus in PNS and CNS. Therefore, animal studies are required to study the mechanisms of virus entry into the brain, its effects on neural function, immune responses, and neurodevelopment 2.

## Immunological responses in Children

**Figure 4:**
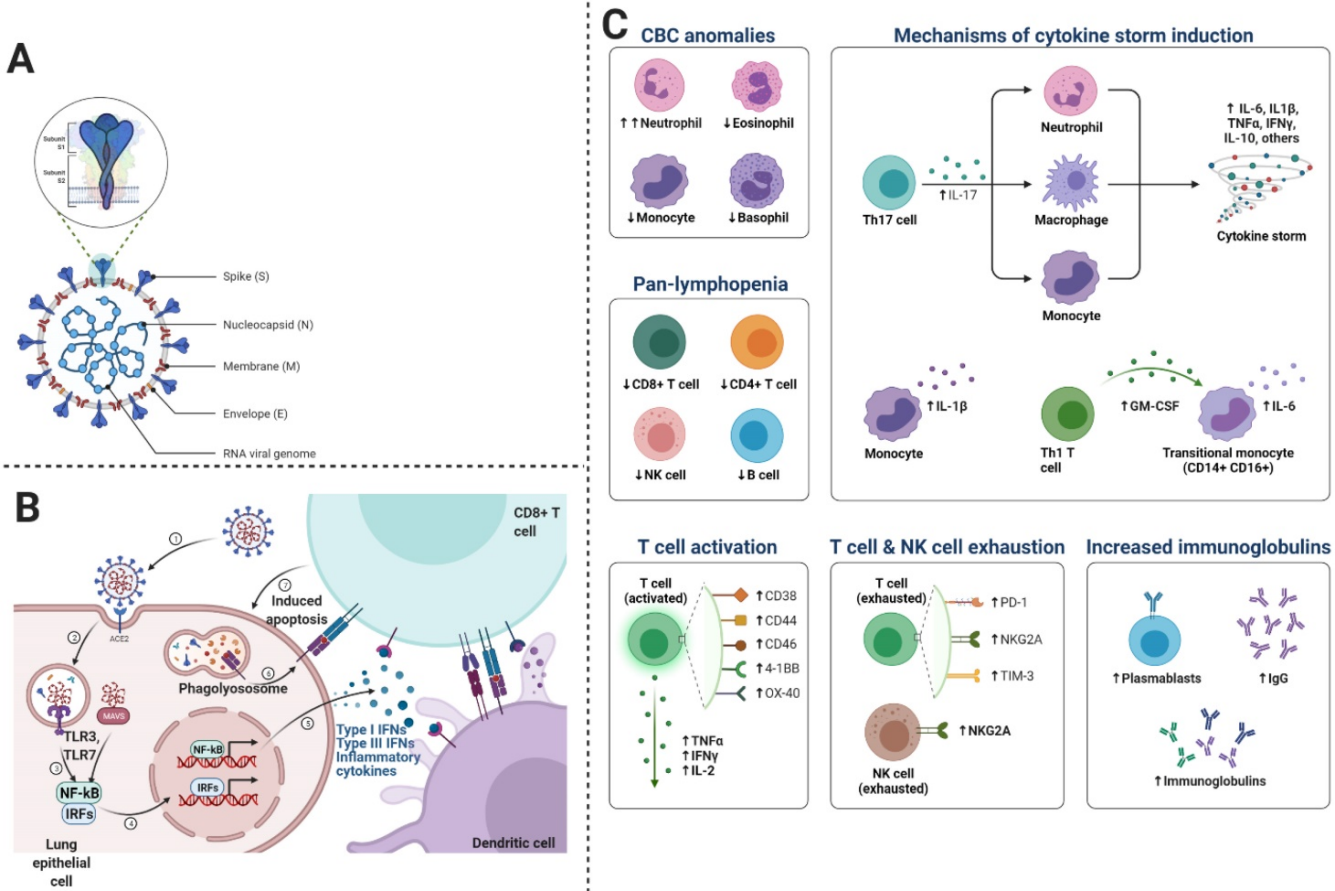
This figure shows the mechanism of immune responses generated after the viral entry into the cell. The virus replicates and induces a massive immune response, which can lead to excessive cytokine release. A- the general structure of SARS-CoV-2, B- Entry mechanism of SARS-CoV-2, and C- immune responses and the involvement of immune cells and molecules in COVID-19.

We know that inflammatory responses in children vary from adults. Such that proinflammatory cytokines are increased with age, thus neutrophil function varies with age. Similarly, the levels of myeloperoxidase, interleukin (IL)-6, IL-10, and p-selectin also increase with age. Since severe COVID-19 disease is characterized by cytokine storm or substantial pro-inflammatory response, thus the risk of ARDS development and multiorgan dysfunction increases 35. In some cases, cytokine expression is not elevated in peripheral blood immune cells, however, type I interferon-induced signatures are strong, indicating the presence of an alternate source other than inflammatory cytokines 36-38. Proinflammatory cytokines associated with SARS-CoV-2 include interferon (IFN)-γ, TNF-α, vascular endothelial growth factor, monocyte chemotactic protein (MCP)-1/C-C motif chemokine ligand (CCL)-2, ILs (IL-1Rα, IL-1β, IL-7, IL-8, and IL-10), granulocyte colony-stimulating factor (G-CSF), basic fibroblast growth factor, granulocyte-macrophage (GM)-CSF, induced protein (IP)-10/CXCL10, macrophage inflammatory protein (MIP)-1α/CCL3, MIP-1β/CCL4, and platelet-derived growth factor 39. During viral replication, SARS-CoV-2 induces the death of infected cells, increases pyroptosis in epithelial cells, IL-1R induces proinflammatory cytokines (IL-6, IFN-γ, MCP-1/CCL2, MIP-1α/CCL3, MIP-1β/CCL4, and IP-10/CXCL) secretion, which in turn recruit T cells and activated macrophages to the infection site. These cytokines induce inflammation and destruction of lung parenchyma. Furthermore, dysregulated IFN signaling influences immunopathology, which leads to impaired viral clearance from infected cells 40. SARS-CoV-2 infection induces CD4 and CD8 T cell response and generates virus-specific antibodies. Patients with ARDS express increased plasmablasts and depleted natural killer (NK) cells, dendritic cells, and CD16+ monocytes. As depicted in Figure 4C, we describe the main cells of the immune system, playing a crucial role in COVID-19.

**Macrophages:** Macrophages involved in COVID-19, are primarily inflammatory monocyte-derived, which express FABP4, ficolin-1 (FCN1), and SPP1 in case of mild and severe disease respectively. The genes expressed by these macrophages encode peripheral monocyte-like markers, chemokines, and inflammatory transcription factors. Moreover, in severe COVID-19, alternative M2 macrophages have been reported to express TREM2, TGFB1, and SPP1, which are profibrotic genes and immunoregulatory genes including A2M and GPR3. These indications suggest that macrophages are not only involved in inflammation but also in pulmonary fibrosis 41,42.

**Natural Killer Cells:** Severe COVID-19 depletes natural killer cells, which exhibit an exhausted phenotype. Natural killer cells are highly depleted in COVID-19 patients with severe disease as compared to those with mild disease. Ventilator-dependent patients exhibit depletion in antiviral cytotoxic CD56dim NK 43. It is not known how the depletion of peripheral natural killer cells occurs; however, it might be due to cell death or the trafficking of cells to infected lung tissue, which is also supported by the expansion of lung macrophages 41. Since COVID-19 leads to the upregulation of TP53 transcription and apoptosis pathways, therefore, cell death may also contribute to the depletion of natural killer cells 43. These details indicate macrophages or cytokines may mediate the dysfunction of natural killer cells in COVID-19.

**T Cells:** Lymphopenia is a common feature of COVID-19, which can be reversed by improved cytokine levels. Lymphocyte count has revealed that CD4+ and CD8+ T cells are significantly reduced in both COVID-19. It is known that there is an inverse correlation between serum cytokine levels and T-cell counts. T-cells may also exacerbate the hyperinflammatory state as COVID-19 patients have been reported with increased pathogenic Th1 CD4+ T cells, which express IFN-γ, IL-6, and GM-CSF 44. These Th1 cells produce proinflammatory cytokines, which play a crucial role in cytokine storm and potentiate tissue damage in the lung. T-regulatory cells are key players in immune homeostasis; thus, their dysregulation can impair immune regulation.

**Neutrophils:** Elevated neutrophil count is prognostic of ARDS and death caused by COVID. In severe cases of COVID-19, neutrophil extracellular traps (NETs) levels increase. NETs are webs of DNA material with oxidant enzymes extruded by neutrophils and antimicrobials to control infections. NETs may induce lung injury and immunothrombosis in patients with COVID-19 42,45. Previous studies based on immunofluorescence reported that neutrophilic infiltrate with numerous Cit-H3+ and MPO+ neutrophils in the lungs. In addition, extracellular DNA lattices with MPO and Cit-H3 have also been reported in COVID-19 patients. It is thought that in pulmonary blood vessels, co-localization of neutrophils with platelets indicates their possible role in microthrombi formation 42. However, further investigations are required to understand the actual role of NETs that triggers platelet aggregation in immunothrombosis.

Although data regarding immune responses to COVID-19 in children are limited, some studies have reported that severe disease in children is associated with obesity. Moreover, high C-reactive protein (CRP) and IL-6 levels have been noticed in children, suggesting that the pro-inflammatory state in children may be similar to that in adults with COVID-19 infection. However, we cannot exclude the possibility of different immune responses. Diorio et al. postulated that SARS-CoV-2 mediated immune activation is associated with endothelial dysfunction 38. Innate and adaptive immune activation mediated excessive inflammation determine the severity of COVID-19, which could be associated with neurological symptoms. MIS-C develops in response to hyperinflammatory responses in genetically susceptible children. Delayed type I and type III interferon (IFN) responses in children with COVID-19 may have higher chances of MIS-C and cytokine storm occurrence. In children, high interleukins and differential B cell and B cell levels have been observed during the acute MIS-C phase 38. S proteins of SARS-CoV-1based antibodies can cross-react, however, COVID-19 has not been reported to be worsened in convalescent plasma receiving individuals. Researchers have reported that immune system hyperactivity is associated with increased inflammatory markers levels. These markers include ferritin, IL-6, erythrocyte sedimentation rate, pro-calcitonin, fibrinogen, D-dimer, and most importantly, C-reactive protein (CRP). On the other hand, high acute innate inflammatory markers levels have also been found linked with neurological complications, because pro-inflammatory cytokines disrupt the BBB, instigate neuroinflammation, and activate glial cells. These phenomena induce neuronal hyperexcitation, fatigue, synapses loss, encephalopathy, and neuron death 9,46-48.

## Conclusions and prospect

Patients (neonatal or children) with COVID-19 are at higher risk of developing severe inflammatory dysregulation, leading to critical illness. Peripheral blood examination for evidence of neutrophil toxic granulations, burr cells, and schistocytes, prior to cytokine profiling may be important for differentiating patients with severe COVID-19 disease from those with MIS-C. This approach can help determine therapeutic approaches based on immunomodulation. Several children have indicated COVID-19 mediated risk of central and peripheral neurological problems such as headache and anosmia, stroke, seizure, and encephalopathy. It seems apparent that COVID-19 affects children and adults differently, which may be attributed to fewer comorbidities in children as compared to adults. Nevertheless, ongoing child's nervous system development indicates that windows of susceptibility are present to neurological injury linked post-infectious mechanisms of COVID-19. Therefore, it is necessary to study associated complications in the pediatric population. Severe COVID-19 disease in children is associated with an inflammatory response mediated by the immune system that may be associated with MIS-C. Pathophysiological pathways could exacerbate the underlying diseases of CNS or PNS via activation of microglia and inflammation. Nevertheless, investigating the long-term impact of COVID-19 on the neurological system is necessary to develop therapeutic options and mitigate serious consequences.

## Figures and Tables

**Figure 1:**
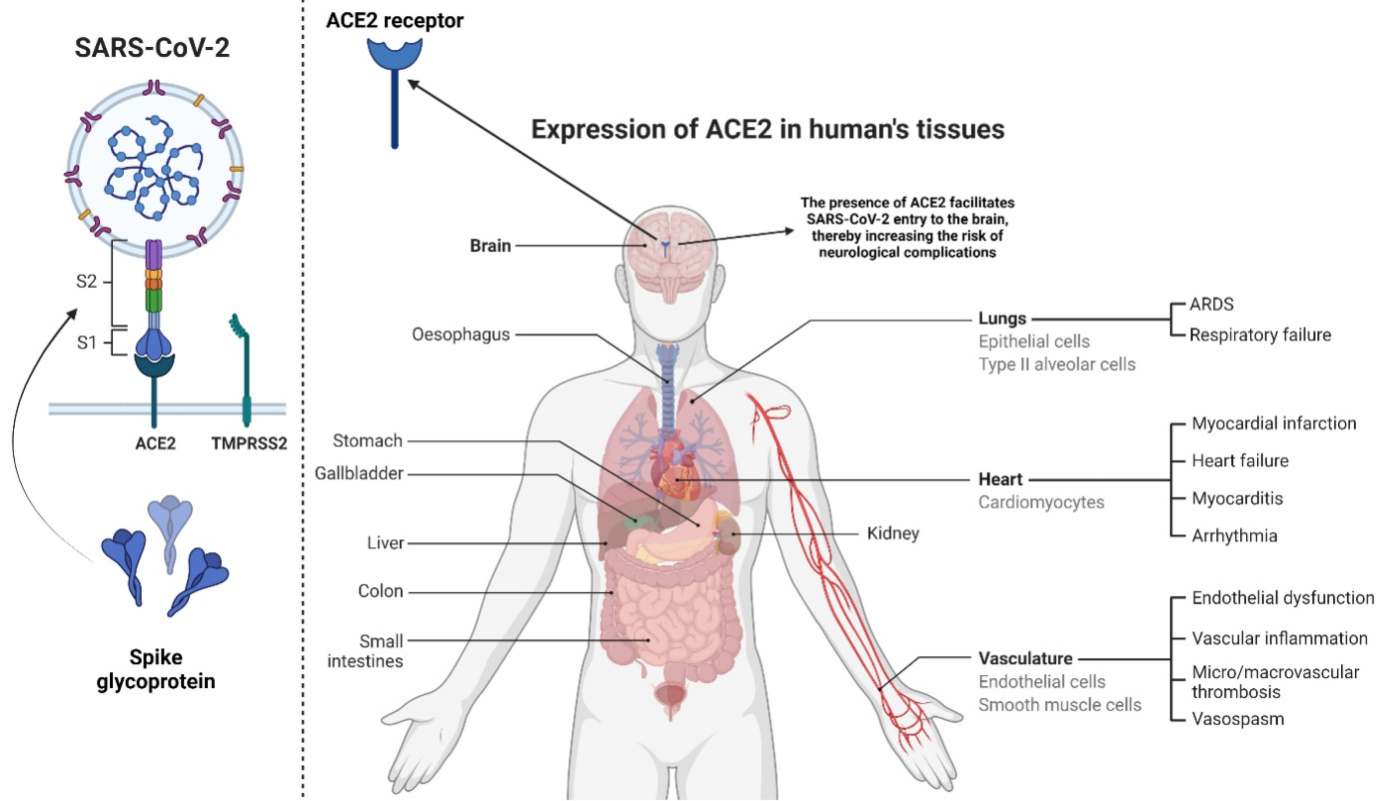
This figure shows the viral structure and spike glycoprotein with its two subunits S1 and S2. Spike protein and TMPRSS2 (left) are essential players in transmission and infectivity. We have also depicted different organs, which can express ACE-2 receptors (Right). They are majorly present in the lungs. Their presence in the brain allows the virus to enter into the brain cells.

**Figure 2:**
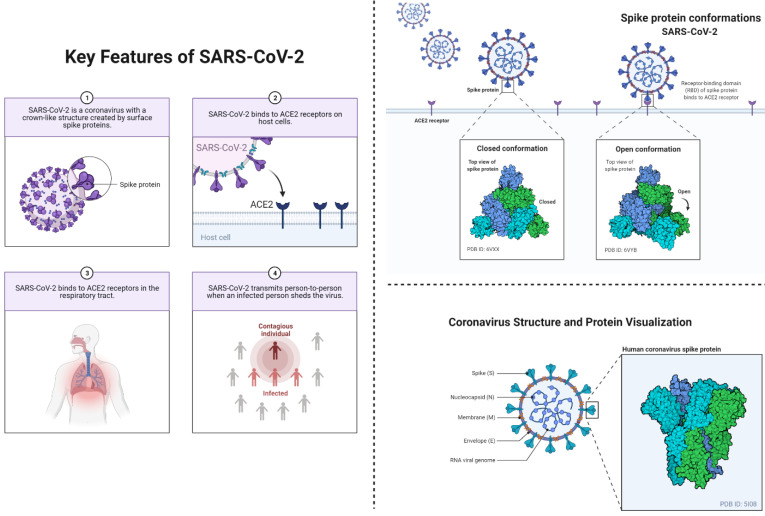
This figure depicts the key features of SARS-CoV-2 (left) including the viral transmission, and entry to the humans. This figure also depicts conformational changes (right) in spike proteins during the infectiousness or entrance to the host cells. The coronavirus spike (S) protein mediates membrane fusion by binding to cellular receptors.

**Figure 3:**
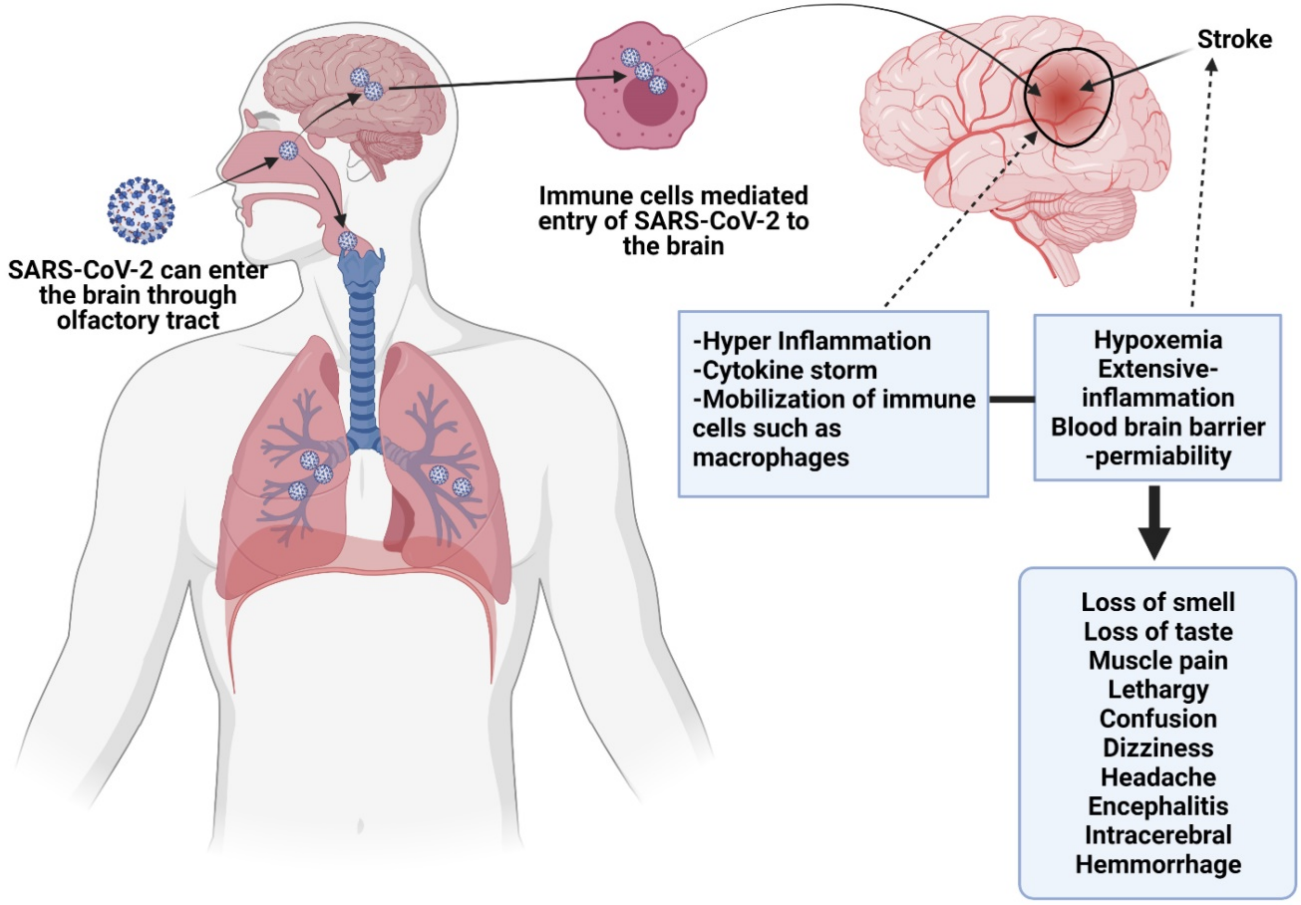
This figure depicts direct viral effects on the nervous system, and endothelial injury. SARS-CoV-2 may cross the blood-brain barrier and enter the CNS via nasal passage or through immune cells transportation. Cytokine release may instigate neurological problems, as well as inflammation induced by SARS-CoV-2 may start autoimmune responses, which can cause demyelination and encephalopathy. SARS-CoV-2 can also induce MIS in children which results in similar inflammatory conditions in children to that of neurological sequelae.
